# Obstetric and Neonatal Outcomes in Pregnancies From a Dedicated Cystic Fibrosis‐Maternal Health Service: A Retrospective Study

**DOI:** 10.1111/1471-0528.70075

**Published:** 2025-11-10

**Authors:** Rebecca Scott, Amy Downes, Ladina Weitnauer, Rebecca Dobra, Natasha Singh, Rachel Robinson, Emily Diable, Saraha Collins, Elaine Bowman, Thomas Tobin, Andrew Jones, Nicholas Simmonds, Imogen Felton

**Affiliations:** ^1^ Department for Obstetric Medicine Chelsea and Westminster Hospital London UK; ^2^ Department of Metabolism, Digestion and Reproduction Imperial College London UK; ^3^ Department of Adult Cystic Fibrosis Royal Brompton Hospital, Part of Guys and St Thomas' NHS Foundation Trust London UK; ^4^ National Heart and Lung Institute Imperial College London UK

**Keywords:** CFTR‐modulators, cystic fibrosis, Elexacaftor/Tezacaftor/Ivacaftor, maternal medicine, pregnancy

## Abstract

**Objective:**

A comprehensive review of maternal, obstetric and neonatal outcomes in pregnancies in females with cystic fibrosis (fwCF) following the introduction of Elexacaftor/Tezacaftor/Ivacaftor (ETI) therapy in a novel, dedicated CF‐Maternal Health service.

**Design:**

Retrospective data review from a CF‐Maternal Health service between September 2020 and February 2025.

**Setting:**

A large adult‐CF service in London, UK.

**Population or Sample:**

Pregnant fwCF attending the Royal Brompton Hospital CF Service.

**Methods:**

Review of CF‐Maternal Health service data.

**Main Outcome Measures:**

Maternal, obstetric and neonatal outcomes.

**Results:**

Fifty‐three fwCF completed 67 pregnancies, with 69 infants born. There were no stillbirths, neonatal or maternal deaths. ETI‐therapy was reported in 81% of pregnancies. In fwCF without CF‐Diabetes, 57% developed gestational diabetes. Hospital admission to treat an infective pulmonary exacerbation was required in 31% of pregnancies. Forty‐five percent of pregnancies delivered vaginally; 78% of babies were born at term. A major congenital abnormality was diagnosed in 4% of infants. Baseline lung‐function correlated positively with birth‐weight and gestation at birth.

**Conclusions:**

FwCF have improved maternal, obstetric and neonatal outcomes since the introduction of ETI‐therapy, within a dedicated CF‐Maternal Health service.

## 1 Introduction

Cystic fibrosis (CF) is an autosomal recessive, multi‐system disease affecting approximately 11 000 people in the United Kingdom (UK) [1]. It results from defective cystic fibrosis transmembrane conductance regulator (CFTR) protein expression due to inherited pathogenic variations in the *CFTR* gene.

Pregnancy rates among females with CF (fwCF) have previously been significantly lower than in the general population [2, 5, 6], with a prevalence of subfertility in 30% of fwCF, and a CF‐associated lower life expectancy [3, 4].

Elexacaftor/Tezacaftor/Ivacaftor (ETI, Kaftrio, Vertex Pharmaceuticals) is a triple‐combination CFTR modulator (CFTRm) drug which improves the underlying cellular defect for > 90% of people with CF (pwCF) [7]. Following ETI introduction, childbirth rates in fwCF have dramatically increased in the US and UK [1, 8]. In the UK in 2019, 58 fwCF had a baby, rising to 140 fwCF in 2022 and 116 fwCF in 2023 [9, 10]. Many factors cause this increase, including direct effects of ETI on the reproductive system, improved general physical health, as well as improved CF prognosis, meaning many fwCF are re‐evaluating life‐ and reproductive‐goals [11, 12, 13].

Historically, CF pregnancies have been considered high‐risk for poor maternal (both CF and obstetric) and neonatal outcomes [14, 15]: 25%–40% infants born preterm, low birth weights, and reports of maternal deaths [14, 16], particularly in those with moderate‐to‐severe preconception lung‐function impairment (percentage‐predicted forced expiratory volume in 1 s (ppFEV_1_) < 60%). Subsequently clinical guidelines stated ppFEV_1_ < 50% and/or coexistent pulmonary hypertension as an ‘absolute contraindication’ to pregnancy [17].

At the Royal Brompton Hospital (RBH)—one of the largest Adult‐CF Centres in Europe—the annual pregnancy rate in fwCF has increased significantly in recent years, from an average of 4 pregnancies per year in 1998–2011 [14], to 7 per year in 2018–20, to 28 pregnancies in 2021. In 2021, a novel, dedicated CF‐Maternal Health Service was established, in collaboration with expert patient‐partners and the local maternal‐medicine centre (Chelsea and Westminster Hospital). This service offers specialist care for fwCF from pre‐conception, through pregnancy and to 4 months post‐partum in a multi‐disciplinary and multi‐professional virtual clinic. The team includes CF‐specialist physicians, nurse specialists, dietitians, physiotherapists and pharmacists, alongside an obstetric physician and obstetrician from the linked maternal‐medicine centre. If the fwCF has her obstetric care at an alternative maternity centre, the obstetricians, obstetrician physicians and midwives from that centre are also invited to join the virtual clinic.

This review describes the maternal, obstetric and neonatal outcomes of fwCF under the care of the Royal Brompton Hospital after the introduction of ETI therapy, in September 2020, to February 2025, and includes those attending the CF‐Maternal Health Service from its inception in September 2021. To our knowledge, it provides the largest and most comprehensive analysis to date of CF‐pregnancy outcomes in England during the ETI era.

## 2 Methods

### 2.1 Data Collection

A retrospective cohort review was conducted using electronic patient records of clinical information and pregnancy outcomes, including from standardised CF Maternal Health clinic proformas, from September 2020 to February 2025.

Due to pre‐existing inter‐individual dosing variability in patient ETI use, detailed subgroups were deemed too small for useful analysis. Therefore FwCF who received any ETI therapy during all or part of pregnancy were considered to have been ‘on ETI’; all others were categorized as ‘no‐ETI’. Lung transplant recipients were excluded from the study. Data collected included baseline demographic information; CF genotype; long‐term medications including ETI use; lung function (spirometry); weight and nutritional status; associated CF complications including pre‐existing diagnoses of cystic fibrosis diabetes (CFD), cirrhosis, pulmonary hypertension, use of non‐invasive ventilation and enteral feeding; mode of conception and whether pregnancy was planned; miscarriages and elective terminations; number of previous pregnancies; hospital admissions for management of CF pulmonary exacerbations; obstetric complications, including gestational diabetes, hypertensive disorders of pregnancy, venous thromboembolism and intrahepatic cholestasis of pregnancy; location, mode and gestation of delivery; neonatal weight and complications. The most recently recorded weight and lung function (spirometry) measures prior to pregnancy were used as a baseline for analysis.

The diagnosis of gestational diabetes was made using a 75 g 2‐h oral glucose tolerance test, performed at 12–14 weeks, and at 24–28 weeks' gestation in line with international CF‐guidelines [18]. Neonatal birth weight centile was calculated using https://timms.le.ac.uk/birth‐weight‐centiles/batch‐calculator.html.

### 2.2 Data Analysis

Data analysis was performed using Prism 10, Graphpad. Descriptive data were used to report outcomes of the study; t‐tests and one‐way ANOVA were used for multiple comparisons.

## 3 Results

During the 53‐month study period, 96 pregnancies were recorded in 68 fwCF. There were 16 first‐trimester miscarriages (including in one fwCF who experienced two miscarriages, and a second fwCF who experienced three miscarriages), resulting in a miscarriage rate of 17% (16/96). There were 13 recorded elective terminations of pregnancy, with none due to foetal abnormalities. Comparing baseline characteristics of fwCF who completed pregnancies (cp) with those who miscarried (mc) there were no statistically significant differences in age (mean age: 30 years [cp] vs. 29 years [mc], *p* = 0.2), baseline absolute volume FEV_1_ (mean FEV_1_: 2.4 L [cp] vs. 2.1 L [mc], *p* = 0.3) or baseline ppFEV_1_ (mean ppFEV_1_: 76% [cp] vs. 70% [mc], *p* = 0.3). Sixty‐two percent (8/13) fwCF who experienced a miscarriage also completed at least one pregnancy resulting in a live birth.

Pregnancies associated with miscarriages and terminations were excluded from the data analysis as on‐going care of these fwCF continued in the general CF clinic, and not the CF‐Maternal Health clinic; therefore, outcomes are reported for the 53 fwCF who completed 67 pregnancies resulting in the birth of 69 babies (including two sets of twin pregnancies).

### 3.1 Baseline Characteristics of fwCF Who Completed Pregnancies

Baseline demographic information and CF‐related health parameters, including ETI‐use in pregnancy, for the 53 fwCF who completed pregnancies, are summarised in Table 1.

**TABLE 1:** Baseline demography, CF‐related health details and reproductive history of a cohort of 53 fwCF who completed 67 pregnancies.

Maternal characteristics	Results
Age at conception, years, median (range)	30 (18–41)
Weight, kg, mean (±SEM)	59.25 (±1.2)
BMI, kg/m^2^, mean (±SEM)	22.7 (±0.4)
FEV_1_, L, mean (±SEM)	2.35 (±0.1)
ppFEV_1_, median (range)	72 (36–136)
FVC, L, mean (±SEM)	3.4 ± 0.1
ppFVC, median (range)	91 (49–140)
Pregnancies affected by CF Diabetes (CFD), % (*n*/*N*)	21 (14/67)
Pregnancies in fwCF requiring enteral feeding for nutritional support of CF‐related low BMI (BMI < 20 kg/m^2^) prior to pregnancy, % (*n*/*N*)	3 (2/67)
Pregnancies in fwCF with cirrhosis, % (*n*/*N*)	1 (1/67)
Pregnancies in fwCF with osteoporosis % (*n*/N)	27 (18/67)
fwCF eligible for ETI therapy % (*n*/*N*)	94 (64/67)
fwCF using ETI therapy prior to pregnancy % (*n*/*N*)	86 (55/64)
fwCF who stopped ETI in the preconception period or during pregnancy % (*n*/*N*)	25 (14/55)
fwCF who restarted ETI during pregnancy	71 (10/14)
Exposure to ETI at any point in pregnancies, % (*n*/*N*)	81 (54/67)
Pregnancies in nulliparous fwCF, % (*n*/*N*)	51 (34/67)
Planned pregnancies, % (*n*/*N*)	72 (48/67)
Pregnancies requiring assisted reproductive technology, % (*n*/*N*)	13 (9/67)

Abbreviations: ETI, Elexacaftor‐Tezacaftor‐Ivacaftor; FEV_1_, volume exhaled at the end of the first second of forced expiration (litres); FVC, forced vital capacity (litres); ppFEV_1_, percent predicted Forced Expiratory Volume in the first second FEV_1_ (%); ppFVC, percent predicted FVC.

Seventeen percent (9/53) fwCF had moderate‐to‐severe lung function impairment (ppFEV_1_ less than 60%). Among these women, two fwCF completed three pregnancies, and a further two completed two pregnancies, leading to a total of 15 infants born to fwCF with a baseline ppFEV_1_ < 60%. No fwCF who experienced pregnancy had pulmonary hypertension or required non‐invasive ventilation before pregnancy.

Fifty‐one percent (34/67) of completed pregnancies were in nulliparous fwCF.

Thirteen percent (9/67) of the pregnancies were conceived via assisted reproductive technology and 28% (19/67) of the pregnancies were unplanned (Table 1).

### 3.2 Maternal Outcomes in Completed Pregnancies

A total of 67 pregnancies in 53 fwCF resulting in 69 live‐births, are included in this analysis.

There were no maternal deaths or intensive care unit admissions of these fwCF during the study period. An in‐patient hospital admission was required for the management of an infective pulmonary exacerbation in 31% of pregnancies (21/67). The pre‐conception mean ppFEV_1_ was significantly lower in those fwCF admitted with pulmonary exacerbations in the antepartum period (mean ppFEV1: 68%) than in those who were not (mean ppFEV1 80%, *p* < 0.05).

Three fwCF developed hypertensive disorders of pregnancy (one case of pregnancy‐induced hypertension; two cases of pre‐eclampsia). One fwCF developed a sub‐segmental pulmonary embolism, and one fwCF, with a preconception history of CF‐cirrhosis, developed intrahepatic cholestasis of pregnancy.

Twelve of the 53 fwCF had pre‐existing CF diabetes (CFD), affecting 21% (14/67) of pregnancies. Excluding pregnancies in females with CFD, 57% (30/53) pregnancies were affected by gestational diabetes.

Mode of birth is summarised in Table 2. Eighty‐three percent (20/24) of fwCF who underwent an induction of labour achieved a vaginal birth (Table 2).

**TABLE 2:** Mode of birth in a cohort of 53 fwCF who had 67 completed pregnancies.

Mode of birth	Results % (*n*/*N*)
Vaginal birth	45 (30/67)
Induction of labour	36 (24/67)
Planned caesarean section	27 (18/67)
Emergency caesarean section	27 (18/67)

### 3.3 Foetal Outcomes

A total of 69 babies were included in this analysis, including two sets of twins. There were no stillbirths or neonatal deaths. Gestation at birth, number of pre‐term births, birth weight and centile are detailed in Table 3.

**TABLE 3:** Characteristics of 69 infants born to fwCF in the study cohort. Birth weight and centile data were unavailable for two infants.

Infant characteristics	Results
Gestation at birth, weeks + days, median (range)	37 + 2 (29^+4^ to 41^+0^)
Preterm births, % (*n*/*N*)	22% (15/69)
Early preterm births (< 34 weeks' gestation), % (*n*/*N*)	4% (3/69)
Birth weight, g, mean (±SEM)	2977 (±62)
Birth centile, median (range)	54th (3rd–96th)

Fifty‐three percent (8/15) of pre‐term births were spontaneous (average gestational age 34 + 6 weeks, range 29 + 4 to 36 + 6 weeks). Of the remaining 7 pre‐term births, six were expedited for respiratory concerns, including in two separate pregnancies for two fwCF. One fwCF, with severe pre‐conception lung function impairment (36% ppFEV_1_) underwent EMCS at 34 + 0 weeks' gestation in her first pregnancy due to an unsustainable decline in lung function; she underwent another EMCS at 34 + 4 weeks' in her 3rd pregnancy, again for a decline in lung function; of note she had a spontaneous pre‐term birth at 35 + 1 weeks' in her second pregnancy. A second fwCF, with moderate‐to‐severe lung function impairment (ppFEV1 50%–60%) with a history of a spontaneous preterm birth at 29 + 4 weeks, had a successful induction of labour at 36 + 5 weeks' having developed significant haemoptysis while taking low‐molecular‐weight heparin for a pulmonary embolism in pregnancy; she then underwent an EMCS at 35 + 0 weeks' gestation in her 3rd pregnancy for an unsustainable decline in lung function. A third fwCF required an EMCS at 33 + 6 weeks' due to complications associated with COVID‐19 infection. She was briefly intubated and mechanically ventilated during the EMCS, and she was extubated without complication directly after birth. A fourth fwCF underwent ELCS at 34 weeks' in early 2021, prior to the establishment of the joint CF‐Maternal Health service; antenatal lung function was stable but standard practice at that time was to advocate iatrogenic pre‐term birth for patients with CF in that obstetric unit. The seventh iatrogenic preterm delivery is reported in a fwCF with GDM, due to concerns about placental insufficiency related to a decline in insulin requirements.

Seven percent (5/69) of infants were large for gestational age (LGA) with birth weight above the 90th centile, of whom four (80%) were born to women with CFD or GDM. Nine percent (6/69) were small for gestational age (SGA) with birthweight below the 10th centile.

**FIGURE 1:**
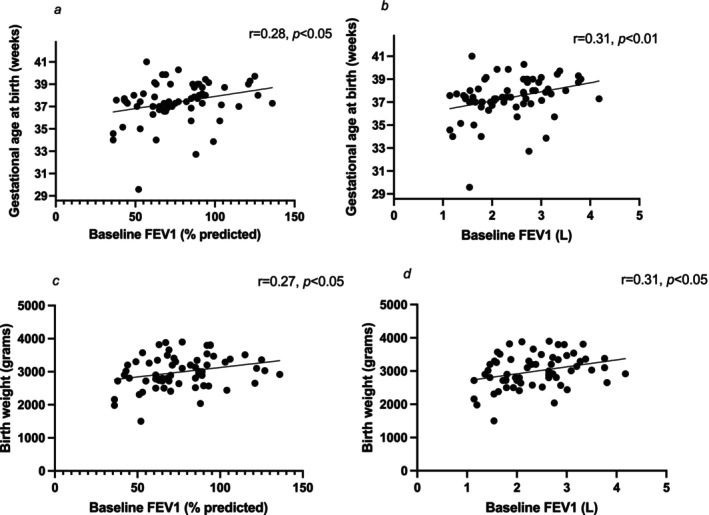
Correlation between (a) baseline ppFEV_1_ and gestational age at birth, and (b) baseline absolute volume FEV_1_ and gestational age at birth (c) baseline ppFEV_1_ and birthweight, and (d) baseline absolute volume FEV_1_ and birthweight.

There was a significant positive correlation between baseline lung function (ppFEV_1_ and absolute volume FEV_1_) with gestational age at birth (*r* = 0.28, *p* < 0.05 and *r* = 0.31, *p* < 0.01 respectively) (Figure 1a,b), and with birthweight (*r* = 0.27, *p* < 0.05; and r = 0.31, *p* < 0.05 respectively) (Figure 1c,d). There was no correlation between baseline BMI and gestational age at birth or birthweight centile (correlation with gestation at birth, *r* = −0.8, *p* = 0.5; correlation with birthweight centile, *r* = 0.2, *p* = 0.12).

FwCF with moderate‐to‐severe pre‐conception lung function impairment (ppFEV_1_ < 60%) were not more likely to give birth prematurely than those with preserved lung function (preterm births: 33% fwCF ppFEV1 < 60% compared to 19% fwCF ppFEV1 > 60%; *p =* 0.75), nor more likely to have a caesarean section (67% of births compared to 50% of births, *p* = 0.38); however, there was a trend for fwCF with moderate‐to‐severe lung impairment to require at least one admission during pregnancy (53% [8/15] compared to 27% [14/52] pregnancies, *p* = 0.07).

Fifty‐eight percent (39/67) of pregnancies received antenatal care in the linked CF‐maternal medicine centre (Chelsea and Westminster Hospital); the remaining pregnancies were managed jointly with local obstetric teams via the CF‐Maternal Health virtual clinic service. There was no statistical difference in gestational age, or in the likelihood of vaginal birth for fwCF, depending on the hospital where delivery took place.

Twenty‐two percent of babies (15/69) were admitted to the neonatal intensive care unit; 47% of these (7/15) were born pre‐term. Ten percent (7/69) of babies developed neonatal jaundice. In total, 4.3% (3/69) of babies were noted to have congenital malformations on antenatal ultrasonography or routine neonatal review: two with cardiac malformations (one case of simple situs inversus, one of pulmonary valve stenosis repaired at post‐natal day two); a third with unilateral moderate hydronephrosis and ureterocoele.

## 4 Discussion

### 4.1 Main Findings

This study provides evidence of improving pregnancy outcomes among a large cohort of fwCF cared for in a dedicated CF‐Maternal Health specialist service in the highly effective CFTR‐modulator era.

Miscarriage rates were not increased compared to the national rate in the UK [19]. There were no stillbirths or neonatal deaths. Over three‐quarters of babies were born at term. There was a marginally elevated risk for congenital anomalies compared to the background UK population (4.3% vs 2.2% respectively) [20], a finding echoed by a recent large French registry‐based study [21], though a lower rate than shown in studies prior to the introduction of ETI therapy [22].

Almost half of all babies were born vaginally, showing that a diagnosis of CF does not preclude a vaginal birth. A higher proportion of CS deliveries was observed than in some studies [5, 21]; this finding may reflect the significant number of multiparous fwCF whose mode‐of‐birth decision may have been influenced by previous CS births prior to the study period, as well as the overall high rate of caesarean birth in the UK at present [23].

The majority of fwCF (81%) continued ETI therapy in part or throughout pregnancy, similar to other recent studies [21, 24]. There were no cases of critical care admission, or of maternal death throughout the follow‐up period, compared to 50% mortality in one previous study from this centre [14]. There was at least one hospital admission for treatment of an infective pulmonary exacerbation of CF in more than a third of pregnancies. As in previous studies, pre‐pregnancy baseline lung function correlates positively with both gestational age and birthweight [14]; nevertheless fwCF with moderate‐to‐severe lung function impairment achieved successful pregnancies with good outcomes. Birth was expedited for respiratory insufficiency in 9% of cases, similar to the pre‐ETI era UKOSS study [5].

### 4.2 Strengths

To our knowledge, this study provides the largest and most comprehensive profile of maternal and neonatal outcomes in CF pregnancies since the widespread availability of the ETI therapy in the UK. The CF Maternal‐Health clinic allows systematic and comprehensive review of pregnant fwCF, incorporating respiratory, obstetric and neonatal information (Appendices S1 and S2). In comparison, a retrospective study from the United States detailed outcomes of 41 pregnancies in fwCF derived solely from information based on CF clinician‐completed questionnaires [25] and lacked objective obstetric and neonatal recorded outcomes. Similarly, a recent large French insurance database study of pregnancies in fwCF lacked the detailed obstetric and neonatal outcome data that helps clinicians and fwCF make informed decisions about maternity care [21].

### 4.3 Limitations

The findings in this study are from a single large Adult‐CF centre, with a novel, dedicated CF‐Maternal Health service which had been developed in partnership with a highly engaged expert‐patient group, as well as maternal medicine networks within London and south‐east England. Outcomes may therefore differ in other centres and settings according to specific CF‐centre size, local pressures and resources. For comparison, a contemporary study from a smaller Scottish CF‐centre showed lower rates of vaginal delivery (31%), and higher rates of pre‐term delivery (31%), but no infants with congenital abnormalities and no admissions for pulmonary exacerbations [24]. Furthermore, all retrospective studies are limited by the quality of the data available; however, an almost complete dataset was achieved.

In this study, three infants with congenital malformations were reported; however only major abnormalities detected on routine antenatal screening and newborn baby checks were recorded. ETI is known to cross the placenta at standard doses [26] and in a US cohort study, 3/26 infants exposed to ETI in utero had evidence of small, subclinical congenital lens abnormalities on ophthalmoscopy examination, which were not identified on newborn light‐reflex checks [27]. Detailed ophthalmic examination is not part of the standard newborn screening in the UK; thus it is possible that neonatal cataracts may be present in this cohort, but not detected. Similarly other malformations not detected by the new born baby check may present later in childhood. There are also in vivo and ex vivo animal studies that suggest in utero exposure to ETI could affect lung and brain development, though a relevant clinical equivalent has yet to be reported [28, 29, 30]. Longitudinal prospective studies are therefore needed to further ascertain drug safety, and determine optimal long‐term follow‐up of infants exposed to ETI in utero. Until then, pregnant fwCF and those planning pregnancy require individualized discussion with their clinicians about the known benefits of ETI therapy, plus the known, and unknown, risks in pregnancy.

### 4.4 Interpretation

There has been a steady improvement in CF‐pregnancy outcomes since the introduction of Ivacaftor, the first CFTR mutation‐specific modulator therapy in 2012 [5, 6, 14, 15, 22]. This current study demonstrates continued improvements following the introduction of ETI therapy. In the present study, the average birthweight centile was 54, with only 22% of infants born preterm. In comparison, a historic cohort from the same associated CF‐maternal‐medicine centre, reported a median birth centile of 31, with 41% of infants born preterm between 1998 and 2001 [14] while the UK Obstetric Surveillance Survey (UKOSS) between 2015 and 2017 reported 38% preterm births in a total of 71 CF‐pregnancies nationally [5].

The last European Guidelines for management of pregnancy in CF were published in 2008, reflecting a legacy of CF care prior to the availability of CFTR modulator therapy. Pregnancy was often considered exceptionally high‐risk and actively discouraged, stating that ‘some (fwCF) will follow medical advice and make a positive decision not to have children’ [15]. Many fwCF of current reproductive age, and non‐CF specialist healthcare professionals were exposed to these historic attitudes, and thus may have lasting and understandable concerns about fwCF pursuing pregnancy. More recent clinical consideration documents reflect the changing clinical landscape, with growing evidence of reassuring pregnancy outcomes [31, 32, 33, 34]. These documents, compiled by CF healthcare providers and fwCF, highlight the importance of shared decision‐making and joined‐up multidisciplinary care between the patient, CF clinicians and maternity teams. This study, based on the RBH schedule of shared CF Maternal Health Care protocol (Appendix S1), demonstrates the benefit of a systematic, coordinated and personalised approach to CF maternity care.

Importantly, fwCF and their healthcare professionals should appreciate that a third of women may experience at least one pulmonary exacerbation during pregnancy that will require hospital admission warranting intravenous antibiotics and focused respiratory physiotherapy. Any hospital admission in pregnancy/post‐partum, in addition to having significant implications on maternal quality of life, has important logistical implications for healthcare professionals as fwCF will need access to medical, obstetric and physiotherapy teams; appropriate pregnancy/breastfeeding‐safe antimicrobials; and isolation/side rooms to prevent lower respiratory tract pathogen cross‐infection which is of paramount importance to CF pulmonary health and long‐term survival.

## 5 Conclusions

This study confirms the significant rise in CF‐pregnancies and provides reassuring data of positive outcomes for both fwCF and infants in the ETI‐era. However, clinical, ethical and research questions in this topic remain. Two prospective observational studies of CF‐pregnancy outcomes are underway—‘Maternal and Foetal Outcomes in the Era of Modulators’ (MAYFLOWERS) [35, 36] in the US; and ‘MATeRnal, InfAnt, Reproductive & Child Health in CF’ (MATRIARCH_CF) [37] in the UK.

The MATRIARCH_CF program is led by investigators from this study group in collaboration with the CF Trust. This UK‐first prospective study of CF‐pregnancy and offspring health aims to definitively counsel women regarding questions raised herein regarding risks and benefits relating to the interplay of pregnancy and CF, in the CFTR‐modulator era.

## Author Contributions

Elaine Bowman, Emily Diable, Rachel Robinson, Saraha Collins, Thomas Tobin: data acquisition/interpretation, revising manuscript; Andrew Jones, Nicholas Simmonds, Natasha Singh, Rebecca Dobra: data interpretation, revising manuscript; Imogen Felton: study conception/design, data acquisition/interpretation, drafting/revising manuscript, final approval of manuscript; Ladina Weitnauer: data acquisition/interpretation, drafting/revising manuscript; Amy Downes: study design, data acquisition/analysis/interpretation, drafting/revising manuscript; Rebecca Scott: study conception/design, data acquisition/analysis/interpretation, drafting/revising manuscript, final approval of manuscript.

## Ethics Statement

Ethical approval was not required for this work, in line with HRA guidance https://www.hra‐decisiontools.org.uk/research/, as it was part of a service evaluation and improvement project. It was registered with the Royal Brompton Hospital Governance and Quality Department.

## Conflicts of Interest

Rebecca Dobra reports grants from UK Cystic Fibrosis Trust, the CF Foundation and Action Medical Research and honoraria for presentations and educational events from Vertex Pharmaceuticals and Chiesi. Saraha Collins has received honoraria from Vertex Pharmaceuticals (Europe) Limited—speaker fees. Nicholas Simmonds has received honoraria for advisory boards from Vertex, Gilead, Pulmocide, Chiesi and Menarini. He has also received honoraria for educational activities from Vertex, Chiesi, Gilead, Teva and Zambon. Imogen Felton has received grants to her institution relating to this work which support Fellowships in CF Reproductive and Maternal Health (Amy Downes and Ladina Waitnauer), by Kings Health Partners Clinical and Academic Innovation Project, and the Royal Brompton Hospital Charity. Unrelated to this work, Imogen Felton has received speaking fees for educational activities from Vertex Pharmaceuticals Incorporated, and a grant from the UK Cystic Fibrosis Trust to her institution (MATRIARCH_CF, SRC 027).

## Supporting information


**Appendix S1:** bjo70075‐sup‐0001‐AppendixS1.docx.

## Data Availability

Data is available by contacting the corresponding author.
