# Supplementary material for: Obstetric and Neonatal Outcomes in Pregnancies From a Dedicated Cystic Fibrosis‐Maternal Health Service: A Retrospective Study
Source: BJOG. 2025 Nov 10;133(4):690–7. doi: 10.1111/1471-0528.70075 (PMC12884204; doi:10.1111/1471-0528.70075)
Supplement: Supplementary file 1 — Appendix S1: bjo70075‐sup‐0001‐AppendixS1.docx. [file BJO-133-690-s001.docx]

**Appendix 1: Royal Brompton Hospital (RBH) CF Reproductive and Maternal Health Multi-Disciplinary Team (MDT) – Monthly Clinic Proforma**

**CF Clinical Nurse Specialist review:**

| **Delivery/due date  (current gestation):** | dd/mm/yyyy  (ww+d) | **Conception method:** | Natural conception / Assisted conception  (eg. IVF/ICSI) |
| --- | --- | --- | --- |
| **Mode of delivery:** | EMCS/PLCS/Vaginal delivery after induction/Spontaneous vaginal delivery | | |
| **Newborn status:** | Boy/Girl (name) | **Newborn weight:** | xxxx g |
| **Obstetric team:** |  | | |
| **Obstetrics' contact info:** |  | | |
| **Previous pregnancy:** |  | **Partner Genetics:** | Not a carrier/carrier for xxx |
| **Clinical update:** |  | | |
| **Pregnancy complications:** | e.g. GDM/HTN in pregnancy/… | | |
| **Delivery complications:** |  | | |
| **Antibiotics in pregnancy:** | **date** – antibiotic (dose, oral vs IV) for pulmonary/other indication | | |
| **Feeding (plan):** |  | | |
| **Contraception:** | After childbirth | | |
| **Occupation and post-delivery plans:** |  | | |
| **Current living Environment:** |  | | |
| **Psychosocial/Family Support or needs:** |  | | |
| **Newborn screening/cord blood:** | IRT result | | |
| **Plan/changes:** |  | | |

**CF Pharmacist:**

| Medications currently taking (Include all CF specific and non-CF specific medications): | DEKAs Plus™ once daily (*NB: water-soluble beta-carotene Vitamin A component, safe in pregnancy*)  ? last levels |
| --- | --- |
| Nebulised and inhaled Therapies   - Mucolytics: - Antibiotics: - Inhalers: |  |
| - Number of course of PO antibiotics in the last 3 months |  |
| - CFTR Modulator therapy? (Drug and start date) |  |
| - If on CFTR modulator therapy when were LFTs done last? |  |
| - Allergy Status: |  |
| - COVID vaccination   (Brand/date) |  |
| - Flu/COVID Vaccination  (Date) |  |
| - Adherence: |  |
| - Any issue with adverse effects |  |
| - Any issue with medication supply/repeat prescriptions? |  |
| - Have medications and their safety in pregnancy/ breastfeeding been discussed? |  |
| - Other medication issues to discuss? |  |
| Plan/ Changes |  |

**CF Physiotherapist:**

| Background  (Wellbeing, sputum, haemoptysis, chest pain, shortness of breath) |  | |
| --- | --- | --- |
| Lung function (Eg. Remote spirometry with Nuvoair^TM^) | **Date completed:** | |
|  | **FEV1:** | **FVC:** |
|  | **Comments:** | |
| Airway Clearance Technique regimen – gestation/post-partum specific? |  | |
| Sinus assessment |  | |
| Nebulisers / inhalation therapy  (Device, equipment needs) |  | |
| CFTR Modulator? |  | |
| Gestation-specific exercise? |  | |
| Musculoskeletal/posture assessment |  | |
| Non-invase ventilation/oxygen therapies |  | |
| Pelvic health assessment |  | |
| **Treatment:** gestation specific / adaptation post-partum / delivery-mode |  | |
| Plan/changes |  | |

**CF Dietitian:**

| *Recent clinical or dietetic issues / concerns:* | - Gastrointestinal Issues: History of distal intestinal obstruction syndrome (DIOS) / constipation? Gastro-oesophageal reflux? - Pancreatic status (pancreatic sufficiency vs. pancreatic insufficiency)? - Diabetes Screening/Management: Diabetes status antenatal / post-partum screening? - Bone health? Last DEXA? |
| --- | --- |
| Nutritional status | Current weight -  Current height -  Previous Weight History:  Weight history during pregnancy  Pre-pregnancy weight and BMI |
| Other issues / Patient Concerns | Appetite?  Calcium containing foods?  Infant feeding? |
| Micronutrients | Supplementation:   - Standard DEKAs Plus™ once daily (*NB: water-soluble beta-carotene Vitamin A component, safe in pregnancy*)   Micronutrient pre-pregnancy:   - Vitamin A, D and E and Iron studies |

**CF Joint Obstetric Medicine and CF Medical:**

| **Lower respiratory tract samples/culture**  -Sputum microbiology  **Spirometry**  ‌-Pre-CFTR-modulator (CFTRm) baseline?  -CFTRm baseline?  -Current?  **Last CT Thorax / CT bronchial angiogram**  -History of haemoptysis?  **Nutrition**  ‌- Pre-CFTRm baseline?  - CFTRm baseline?  - Current?  - Diabetes status / control?  **CF Antenatal / Postpartum Considerations**  -Shared decision for continuing/stopping CFTRm?  -Local/Category B or Linked Category C Maternal Medicine Hub maternity care setting?   - Joint outreach clinic? Intravenous access / port-a-cath? - Screening echocardiography to exclude pulmonary hypertension if ppFEV<40% and/or on chronic non-invasive ventilation due to type 2 respiratory failure?   -Antibiotic allergies/intolerances?  -Plan for choice of antibiotics in the context of a pulmonary exacerbation?  -History of haemoptysis? Need/risk for prophylactic anticoagulation/anti-aggregation therapies?  -Mode of iron supplementation (risk of severe constipation with oral supplements)? |
| --- |

**Appendix 2: RBH Schedule of Co-ordinated CF-Maternal MDT Care**

**
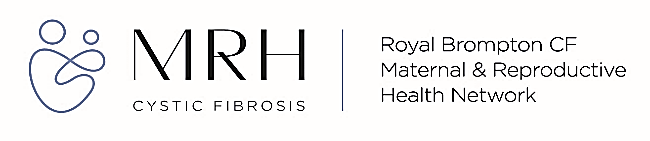
**

| **Timeline** | **CF MDT Care Considerations** | **CF-Diabetes targets** | **Gestational diabetes** | **Obstetric** |
| --- | --- | --- | --- | --- |
| ***Pre-conception***  ***(~12months before trying to conceive)*** | **CFTR gene mutation screening in partner**  Assess maternity care setting (B or C)  Assess psycho-social needs/ support /parenthood impacts  Baseline tests (iron + vitamins ADEK, HbA1c, DEXA)  Baseline thoracic imaging and lower respiratory tract microbiology  Special considerations if history of haemoptysis/bronchial artery embolization, non-tuberculous mycobacteria or fungal infection  Baseline echocardiography if ppFEV1 <40% to screen for pulmonary hypertension  Discuss individual considerations & evidence re: CFTRm choice | HbA1c <6.5% or 48mmol/mol |  | Assess assisted conception needs (for infertility or pre-implantation diagnostics)  Liaison with fertility clinic  Genetic counsellor if partner is a CFTR mutation carrier |
| *By 11 weeks* | **Monthly CF & Obstetric Medicine**  **Joint Hybrid Virtual / FTF Clinic**  *-Updates in emerging evidence base and gestation specific physio*  *-Assess vitamin ADE and iron studies*  *-Outreach Maternity Team liaison* | HbA1c <6% 42mmol/mol  TIR: 70% 5.3-7.8mmol/l  Fasting <5.3mmol/l  1hr post prandial <7.8mmol. | If previous GDM: OGTT | Book in with appropriate maternity team  Dating scan |
| *12-14 weeks* |  |  | 1^st^ OGTT | Appropriate GDM monitoring and Rx (CF-nutrition goals aware) |
| *16-20 weeks* |  | Retinopathy screen |  | Anomaly scan |
| *28 weeks* |  |  | 2^nd^ OGTT | Fetal growth scan |
| *32 weeks* |  |  |  | Fetal growth scan |
| *36 weeks* | Consider choice of antibiotics if pulmonary exacerbation (elevated risk for necrotising enterocolitis if exposed to co-amoxiclav)  Pack CF bag! | Plan induction | Plan induction | Fetal growth scans  Pack baby bag |
| *38 weeks* |  | Induce by 38+6 | Induce by 38+6 |  |
| *40 weeks* |  |  |  | Low intervention active birth  Avoid carboprost (bronchospasm) |
| *4, 8, 16 weeks postpartum* | Airway clearance  Psychosocial support  If breastfeeding, ensure nutrition +micronutrients support  If infant had been exposed to ETI , refer for ophthalmic screens  Maternal-Infant dyad review 4 - 8 weeks at RBH |  | 3/12 HbA1c | Newborn screen result, contraception, pelvic floor |
